# Mosses as Biomonitors of Atmospheric Trace Metal and Nitrogen Deposition: Spatial Distribution and Temporal Trend in Yancheng, China

**DOI:** 10.3390/plants14091315

**Published:** 2025-04-26

**Authors:** Xiaoli Zhou, Jing Li, Peng Yan, Nana Lu, Linyu Lu, Qian Ni, Junrong Zhang, Yanming Fang

**Affiliations:** 1 School of Marine and Biological Engineering, Yancheng Teachers University, Yancheng 224002, China; zhouxiaoli0404@163.com (X.Z.); 15251897336@163.com (J.L.); yanpengsyjsq@163.com (P.Y.); lunana0621@163.com (N.L.); 15251069766@163.com (L.L.); 19518893186@163.com (Q.N.); 13270045660@163.com (J.Z.); 2 Northeast Institute of Geography and Agroecology, Chinese Academy of Sciences, Changchun 130102, China; 3 Co-Innovation Center for Sustainable Forestry in Southern China, College of Life Sciences, Nanjing Forestry University, Nanjing 210037, China

**Keywords:** atmospheric deposition, moss biomonitoring, trace metals, nitrogen, geographic information system (GIS), positive matrix factorization (PMF) model

## Abstract

This study assessed air quality in Yancheng, China, using moss biomonitoring. The moss species, *Haplocladium microphyllum* was chosen, and mosses were collected from 67 sites across Yancheng during July and August 2022. The concentrations of Al, Co, Cr, Cu, Fe, Mn, Ni, Pb, Zn, V, and nitrogen in mosses were determined, and the spatial distribution and temporal trends of atmospheric trace metals and nitrogen deposition in Yancheng were explored by comparing the current data with that of a similar study conducted in 2017. In 2022, high concentrations of metals and nitrogen in mosses were found in northern and southwestern Yancheng, whereas lower concentrations were observed in southern and southeastern Yancheng for metals and central Yancheng for nitrogen. Since 2017, the moss concentrations of Zn, Cu, Ni, and Cr have increased, while that of V has declined, with no notable changes observed in other metals and nitrogen. Contamination factor analysis indicated that Pb and Cu contamination levels escalated from moderate and slight (2017) to severe and moderate (2022), respectively. The Positive Matrix Factorization (PMF) model identified five dominant contamination sources of metals and nitrogen in 2022 mosses: natural source (21.4%), traffic emission (17.84%), fuel combustion derived from coal and heavy oil (22.71%), agricultural activities (19.37%), and industrial activities (18.68%). This study highlights the significance of moss biomonitoring, along with data analysis and emission source inventories, as essential tools for evaluating air quality in Yancheng.

## 1. Introduction

Air quality represents a global challenge influenced by diverse atmospheric contaminants, including particulate matter, hazardous inorganic compounds, heavy metals, reactive nitrogen (N), and persistent organic pollutants [1]. The atmosphere serves as a medium for the transport of both organic and inorganic substances originating from both natural and anthropogenic sources. The primary anthropogenic sources of metals and reactive N in the air include fine soil dust, emissions from metal processing, vehicle exhaust, fossil fuel combustion, agricultural fertilizer application, and livestock farming [2,3].

Wet and dry deposition are crucial processes that remove pollutants by transferring them to terrestrial ecosystems [4]. The long-term accumulation of atmospheric metals in ecosystems poses substantial risks to environmental integrity and public health, mainly through inhalation and the food chain [5,6]. Elevated metal concentrations in the air we breathe are linked to a range of acute and chronic health conditions in humans. In addition, excessive nitrogen deposition can harm biodiversity, disrupt plant nutrient balance, alter soil and water composition, and cause risks to human well-being [7]. For these reasons, it is essential to monitor metal and nitrogen deposition regularly to safeguard vulnerable ecosystems and identify high-risk areas.

Naturally growing mosses are recognized as effective biomonitors for atmospheric trace metal and nitrogen deposition, demonstrating extensive and long-term use in environmental monitoring programs across Europe [8]. Since 1990, the European moss survey has been conducted at five-yearly intervals. According to the latest available published data from the 2015 survey, 36 countries reported trace metal concentrations in mosses, with approximately 12 countries additionally reporting nitrogen concentrations [9]. The principle behind using mosses to measure atmospheric trace metal and nitrogen deposition lies in the fact that carpet-forming pleurocarpous mosses lack true roots and vascular systems. As a result, they primarily absorb trace elements and nutrients directly from precipitation and dry deposition while minimizing the uptake of metals from the substrate [10]. The concentrations of trace metals and N in mosses offer an alternative, time-integrated method for assessing the spatial distribution and temporal trends in the deposition of these substances from the atmosphere to terrestrial ecosystems [11]. Analyzing the elemental concentrations in mosses is simpler and more cost-effective than traditional deposition methods, as it eliminates the need for numerous deposition collectors. This allows for a significantly higher sampling density when appropriate moss species are chosen compared to conventional deposition analysis [12].

Multivariate statistical and geostatistical techniques can be employed together to assess the spatial distribution patterns and source identification of trace metals and nitrogen accumulation in mosses [13]. The receptor model, a widely used method for source apportionment in moss metal and nitrogen analysis, aids in qualitatively distinguishing contamination sources in mosses (as receptors) and quantitatively estimating the contribution of each specific source. The Positive Matrix Factorization (PMF) receptor model, a cutting-edge method in source resolution, has been effectively applied in apportioning source contributions in mosses, sediments, and soils in recent years [14,15]. In this study, the PMF model was utilized to identify the sources of trace metals and nitrogen in mosses collected from Yancheng, China.

In 2017, atmospheric deposition of trace metals and nitrogen in Yancheng was investigated using moss biomonitoring. The northern and southwestern regions of Yancheng were identified as ‘*hotspots*’ of metal contamination, while the central area of Yancheng was recognized as a zone of high nitrogen deposition [16,17]. This study presents the metal and nitrogen concentration data collected from the 2022 moss survey in Yancheng. The aim of this research is to assess air quality in Yancheng, with the following objectives: (1) evaluate atmospheric metal and nitrogen deposition in Yancheng using moss biomonitoring; (2) examine the spatial distribution and temporal trends of metal and nitrogen concentrations in mosses since 2017; and (3) identify the potential factors influencing metal and nitrogen concentrations in the 2022 moss samples.

## 2. Results

### 2.1. Metal Concentrations and Contamination Scale in 2022

The concentrations of trace metals in the moss samples from Yancheng are summarized in Table 1, with site-specific geolocation and elemental profiles provided in Table S1. The concentrations (in mg/kg) of these elements, along with their means and standard deviations, are as follows: Al (5416 ± 2642) > Fe (4517 ± 2300) > Zn (995 ± 701) > Mn (171 ± 72.5) > Cu (42.2 ± 34.1) > Cr (39.4 ± 23.7) > Pb (29.3 ± 36.9) > Ni (17.0 ± 11.3) > V (9.66 ± 3.74) > Co (4.01 ± 1.38). The concentrations of Al and Fe exceeded those of other elements by one to three orders of magnitude. The concentrations of Pb and Cu exhibited pronounced disparity, characterized by high coefficients of variation (CVs > 75%, Table 1), whereas other elements demonstrated greater stability, with moderate CVs ranging from 25% to 75%. The median concentrations of metals in mosses collected from Yancheng, China (2022), were compared with data from Mongolia, a neighboring country, and Norway, representing a clean area in Europe (Table 2). The concentrations of multiple metals in mosses from Yancheng were significantly higher than those in Mongolia and Norway, especially Zn, Ni, Pb, and Cr, while the concentrations of Fe and Al were similar to those in Mongolia.

**Figure 1:**
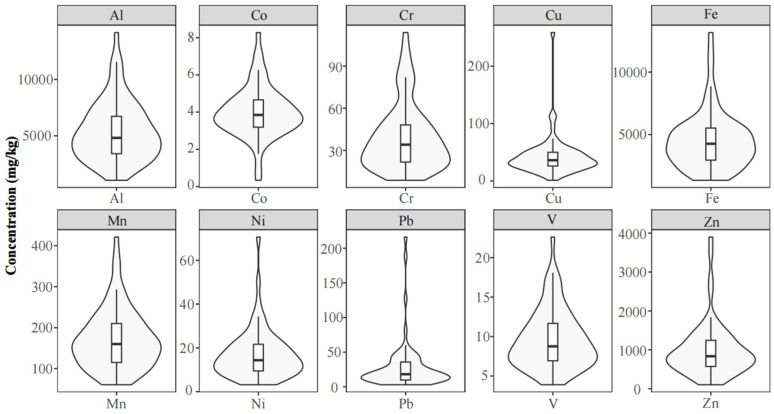
Violin plots of trace metals in mosses from Yancheng, China. The vertical bands, thin vertical lines, and shapes on the sides represent quartile range, variation range, and approximate frequency distribution of the data, respectively.

The violin plot, integrating features of a boxplot and kernel density estimation, was employed to visualize the median, variation, and distribution of trace metal concentrations in moss samples from Yancheng (Figure 1). The thin horizontal line represents the median, while the box extends from the lower quartile to the upper quartile. The characteristic silhouette of the violin plot reflects the probability density of the data, with wider sections indicating higher data density. The patterns for Pb, Cu, Ni, and Zn in mosses showed considerable variation (Figure 1).

The contamination factor (CF) values for all elements were quantified following established protocols [18] (see Table 3), and their corresponding contamination levels were evaluated. The CF of Pb indicated severe contamination, while CFs for Zn, Cu, Ni, V, Fe, Mn, Al, and Cr suggested moderate contamination. The CF for Co demonstrated slight contamination. Similar CF values were observed for Zn, Ni, Fe, Mn, Cr, and V in both the current data and the 2017 data [17]. The contamination levels of Pb and Cu in mosses increased from moderate and slight contamination in 2017 to severe and moderate contamination in 2022, respectively.

### 2.2. Spatial Patterns of Trace Metals in 2022 and Temporal Trends Since 2017

**Figure 2:**
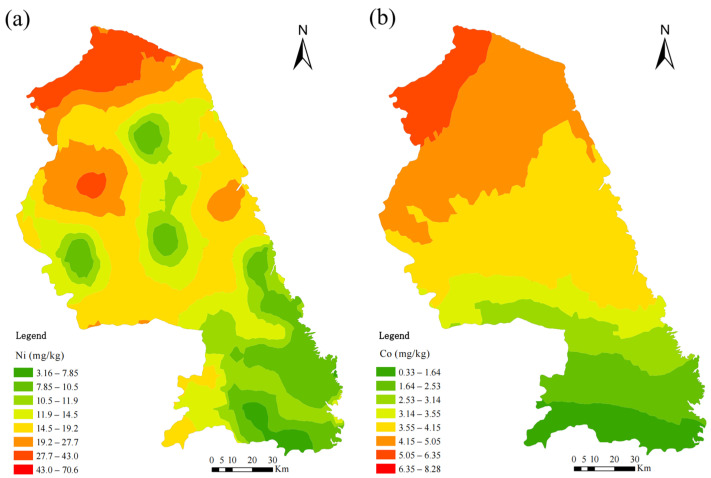

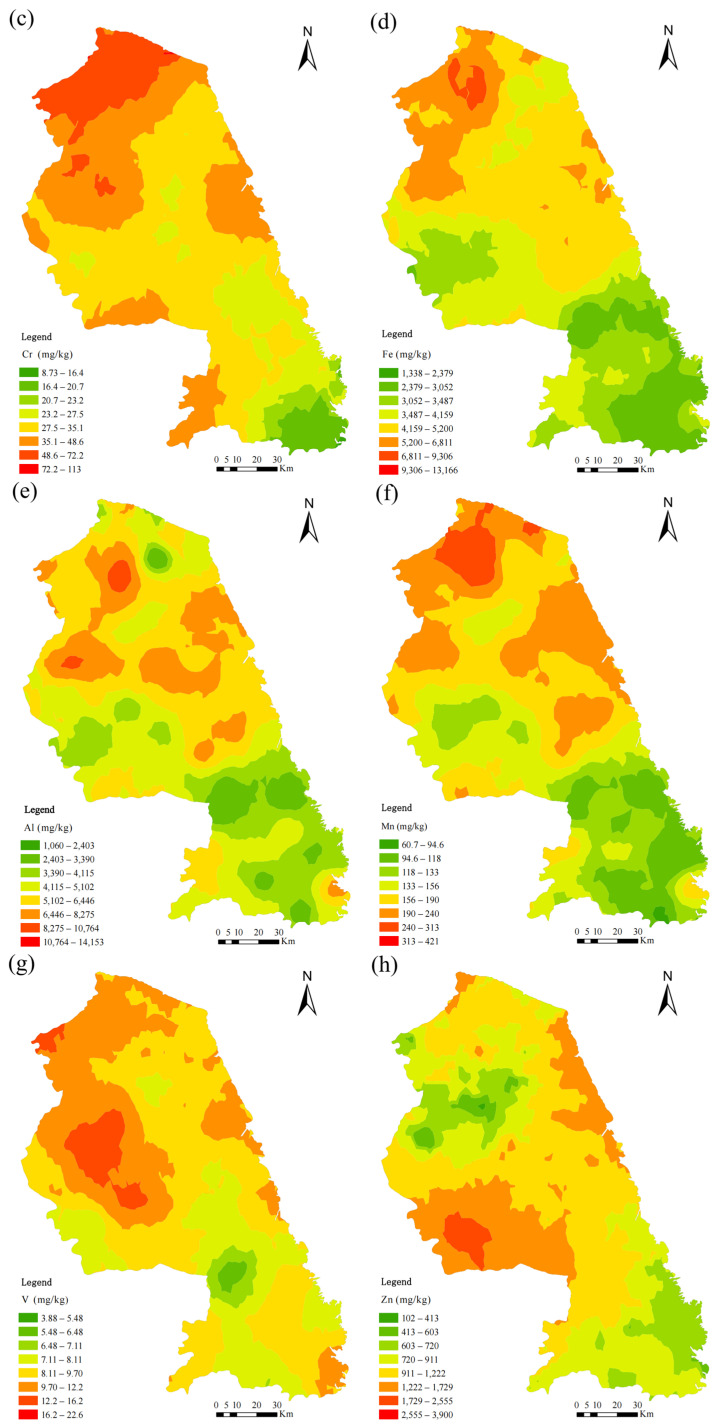

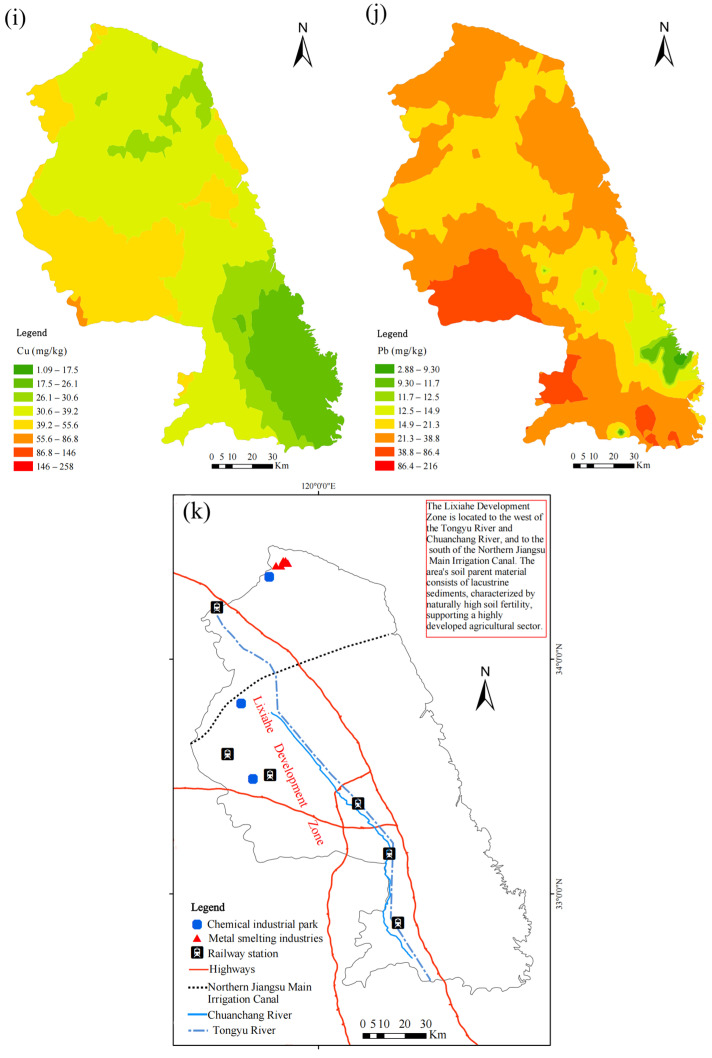
Spatial distribution of trace metal concentrations in mosses (2022; **a**–**j**) and industrial zones, infrastructure, water systems, and transportation networks in Yancheng (**k**).

The spatial patterns of trace metal concentrations in mosses from Yancheng in 2022 are shown in Figure 2a–j. The spatial distribution of moss trace metals in 2022 was similar to that in 2017, with lower concentrations observed in southern and southeastern Yancheng. Generally, higher levels were found in the northern and southwestern regions, resulting in a southeast-to-northwest gradient for many metals in 2022. The metals Cr, Ni, Co, and V showed an enrichment in the north of the study area. Elevated concentrations of Al, Fe, and Mn were observed in the northeastern region, while the highest concentrations of Zn and Cu were found in the southwestern region. The distribution pattern of Pb differed from the other elements, with relatively high concentrations throughout the study area, peaking in the southwestern and northeastern regions.

The 2022 and 2017 moss survey data were compared to assess temporal trends in metal concentrations in mosses from Yancheng. The statistical distributions of the data from both years were similar, mostly log-normal, allowing the Mann–Whitney U Test to be used to detect significant differences (*p* ≤ 0.05) between the two years for each element. The concentrations of Fe, Mn, and Pb exhibited no statistically significant differences (*p* > 0.05) when comparing measurements from 2017 and 2022. However, significant increases (*p* < 0.05) in metal concentrations were observed, with Zn rising by 522%, Cu by 72%, Ni by 64%, and Cr by 174%. Conversely, a significant decrease (*p* < 0.05) of 36% was noted for V. Despite these significant changes in concentrations, the contamination levels (except Cu) remained unchanged (Table 3). Although Pb concentrations increased by 26% in 2022 compared to 2017 (a non-significant increase), this, along with the rise in Cu concentrations, contributed to an overall increase in the metal contamination level (Table 3).

### 2.3. Spatial Patterns of Nitrogen in 2022 and Temporal Trends Since 2017

In 2022, the N concentrations in mosses ranged from 0.85% to 3.81%, with an average value of 1.67 ± 0.53%. According to the estimation by Pitcairn et al. [19], moss N content reaches 2% when exposed to an annual N deposition rate of 20 kg ha^−1^. This threshold suggested that atmospheric nitrogen input in Yancheng likely remained under 20 kg N ha^−1^ yr^−1^ during the study period. 

**Figure 3:**
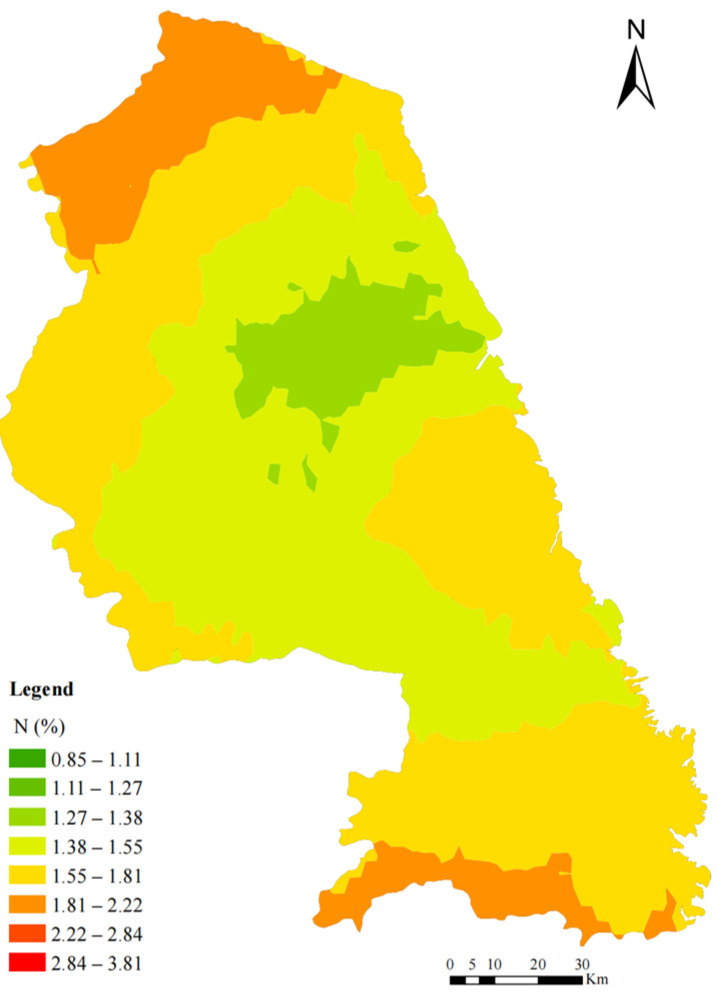
Spatial patterns of nitrogen (N) concentrations in mosses in Yancheng, 2022.

Since 2017, the mean N concentration in mosses has not shown a significant change (a decrease of 0.28%); however, the spatial distribution pattern has shifted over time. In 2022, elevated N concentrations in mosses were observed in the northern, southwestern, and eastern coastal regions of Yancheng, displaying a gradual increase from the central area toward the northern, southern, and eastern zones (Figure 3). In contrast, the 2017 data revealed three N hotspots, primarily in central Yancheng, with a distinct west-to-east decreasing gradient [16].

### 2.4. Source Appointment of Trace Metals and Nitrogen in 2022

**Figure 4:**
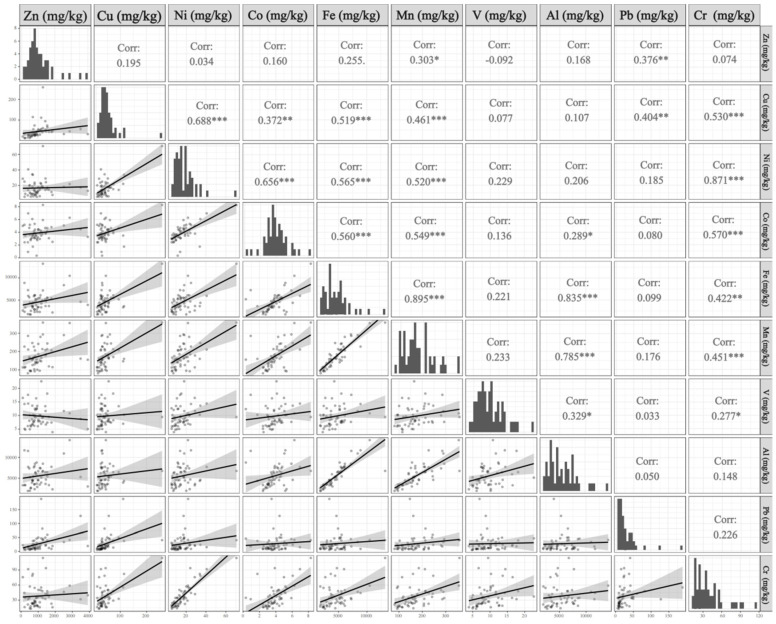
Correlation coefficients between different trace metals in mosses in Yancheng, 2022. Note: * *p* < 0.05, ** *p* < 0.01, *** *p* < 0.001.

The Spearman correlation coefficients for ten trace metals are shown in Figure 4. Strong positive correlations were found between Mn and Al, Mn and Cr, Mn and Fe, Mn and Co, Co and Cr, Co and Fe, and Fe and Al (*p* < 0.001). Additionally, Ni, Fe, Mn, Cr, and Mo exhibited close relationships with Cu (*p* < 0.001), while Co, Fe, Mn, and Cr were also highly correlated with Ni (*p* < 0.001). To further investigate the relationships between these metals, as well as their connection to nitrogen and potential pollution sources, a more detailed Positive Matrix Factorization (PMF) model was employed.

**Figure 5:**
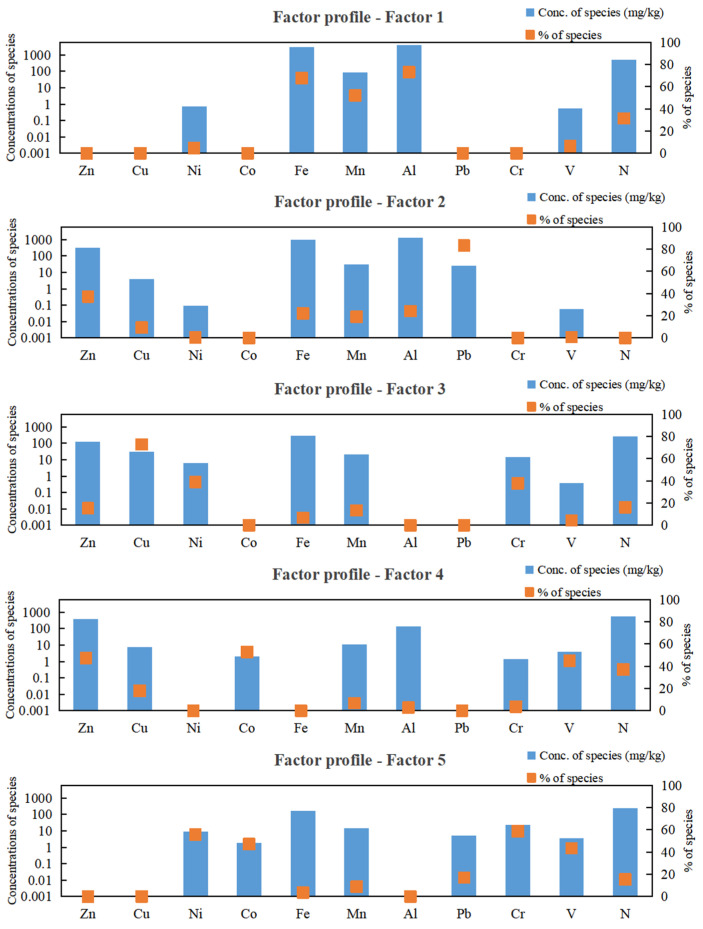
Factor profiles from PMF model using trace metal and N concentrations in mosses in Yancheng, 2022.

The analytical datasets for the EPA PMF 5.0 model comprised concentrations of ten metals and nitrogen in 67 moss samples, paired with their measurement uncertainties. Principal component analysis (PCA) was employed to identify the optimal number of components accounting for 70–80% of the cumulative variance. The results revealed that elements in Yancheng mosses could be effectively reduced to 4–5 principal components (Table S3). To refine the PMF model performance, factor numbers were systematically evaluated at both 4 and 5 levels. Additionally, the ‘random start seed number’ option was selected, and the number of runs was set to 20. The optimal solution was achieved at five factors, characterized by minimized and stabilized *Q*-values. Moreover, scaled residuals of 95% of samples fell within the range of −3.0 to 3.0 (Table S4), and the measured and simulated concentrations of all elements exhibited strong agreement, with the coefficients of determination (R^2^) for the fitted regression curves exceeding 0.75. Following the selection of the optimal number of factors through base model analysis, the rotational tool was applied to the model to reduce collinear sources, thereby obtaining the optimal factor contribution rates for source apportionment (Figure 5). The displacement (DISP) analysis revealed zero swap counts across all tested d*Q*_max_ levels (4, 8, 15, 25) (Table S5), demonstrating little rotational ambiguity in the model solutions.

As illustrated in Figure 5, the PMF model identified five dominant factors influencing the concentrations of metals and nitrogen in 2022 mosses. Factor 1 was predominantly loaded on Al (73.09%), Fe (67.63%), and Mn (52.14%); Factor 2 was primarily characterized by Pb (83.10%) and Zn (37.29%); Factor 3 was weighted on Cu (72.80%), Ni (39.06%), and Cr (37.75%); Factor 4 was defined by Co (52.89%), Zn (47.45%), V (44.87%), and N (37.03%); and Factor 5 was primarily influenced by Cr (58.69%), Ni (55.82%), followed by Co (47.11%) and V (43.45%). Based on the factor fingerprints of each element, the overall percentage contribution of each factor was calculated. Factor 5 accounted for the largest share (22.71%) of trace metals and nitrogen in the atmosphere in Yancheng, followed closely by Factor 1 (21.4%), Factor 4 (19.37%), Factor 3 (18.68%), and Factor 2 (17.84%).

## 3. Discussion

### 3.1. Metal Concentrations and Contamination Scale in 2022

The observed concentration disparities in mosses between Yancheng and other regions likely reflected variations in local emission profiles from both natural and anthropogenic sources. Elevated Fe and Al levels in Yancheng and Mongolian mosses may originate from Asia’s crustal enrichment background, which affects the chemical composition of soil dust [1]. Different moss species employed across study areas could introduce interspecific bias in metal accumulation capacities, potentially compromising comparative analyses [11,20]. Standardized protocols utilizing conspecific mosses are, therefore, recommended for biomonitoring comparability. The high CV (>75%) values for Pb and Cu suggested that their concentrations were affected by various factors. The violin plots showed that the concentrations of Pb, Cu, Ni, and Zn in the mosses exhibited significant variation, which was consistent with the results of the CVs (Table 1), indicating potential anthropogenic inputs for these elements in Yancheng [20]. For CFs, the observed differences for Pb and Cu were primarily due to variations in the concentrations (*C_i_*) of these metals in mosses between 2017 and 2022, as the calculation method for background concentrations (*BC_i_*) was consistent between the two years.

### 3.2. Trace Metals—Spatial Patterns in 2022 and Temporal Trends Since 2017

**Figure 6:**
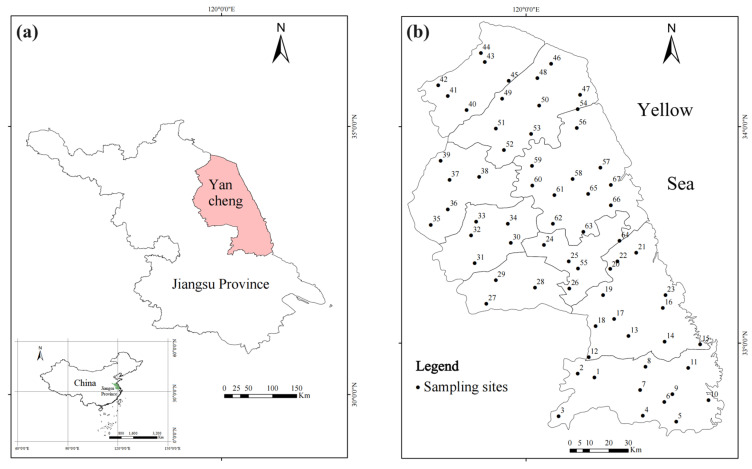
Sites where mosses were sampled for trace metals and nitrogen analysis in 2022. (**a**) Jiangsu province, where the Yancheng region is the sampling area, is outlined. (**b**) Locations of the sampling sites across Yancheng.

In our study, the spatial patterns of metals could be explained by local emission sources and meteorological factors. Elevated metal concentrations in mosses aligned spatially with proximity to anthropogenic activities. The elements associated with geogenic sources (Cr, Ni) and metal processing activities (Cr, Ni, Co, and Cu) are concentrated in the northern Yancheng, which is characterized by the developed steel smelting industry, particularly of Ni-Fe smelting (Figure 2k). The high concentrations of Al, Fe, and Mn observed in the northeastern part of the city likely resulted from rock weathering, as these elements are abundant in the Earth’s crust [21,22]. The highest concentrations of Zn, V, and Cu were found in the southwestern region, which is the primary agricultural area of Yancheng (Figure 2k), indicating that the sources of Zn, V, and Cu may be agricultural emissions [5]. The wind direction frequency patterns (Figure S1) revealed predominant southeast/northeast airflow during the study period (2022), facilitating atmospheric transport of pollutants to downwind northern and southwestern sectors, consistent with observed metal enrichment patterns in mosses (Figure 2a–j). While northeast/southeast marine winds may convey oceanic elements (Na, K) inland from the adjacent Yellow Sea (Figure 6b), their impact on the metal concentrations observed in this study was relatively minimal. In winter, the northwest wind (Figure S1) may carry metals emitted by inland industries, but due to the weaker wind intensity, the influence on the concentrations and distribution of metals in the mosses was limited.

The absorption of inorganic elements by mosses is influenced by a range of factors, including the physicochemical properties of metals, their uptake mechanisms, and their bioavailability during deposition [23], which can result in significant variations in metal concentrations. Additional factors such as cation competition [24], wind-blown mineral dust, precipitation chemistry, and varying levels of natural and anthropogenic emissions [25] further contribute to these variations. These influences are primarily linked to meteorological conditions, which affect both the dispersion and chemical behavior of atmospheric pollutants [26,27]. Wet and dry deposition, including rain scavenging and gravitational settling, are key pathways through which metals are transferred from the atmosphere to terrestrial ecosystems [28]. From June to August 2017, Yancheng experienced intense rainfall events [29], while in 2022, precipitation was more evenly distributed but significantly reduced (Figure S2). Under varying rainfall patterns but with consistent emission inventory and sources during the sampling and moss-growing periods, we proposed that weather conditions were likely the key drivers behind the significant differences in moss metal concentrations observed in Yancheng between 2017 and 2022. The decrease in V levels in 2022 may be linked to its tendency to bind with atmospheric fine particles (PM_2.5_ or smaller), which remain suspended in the air [30], thus limiting its absorption by mosses through dry deposition. Reduced precipitation in 2022 likely also limited the wet deposition of V on moss samples [31]. The observed differences in concentrations of Zn, Cu, Ni, and Cr between the two years may also be attributed to the climatic factors affecting atmospheric deposition, as reflected in the accumulation of these metals in mosses [32,33]. In particular, dry deposition, which is the primary pathway for metals like Pb, Cu, and Zn to be absorbed by mosses, played a dominant role in 2022 due to the lower precipitation [34]. In contrast, Yancheng experienced higher precipitation in 2017, especially in the sampling months of July and August, which had the highest rainfall of the year (Figure S2). The observed lower concentrations of trace metals in 2017, particularly for soluble elements such as Cu and Zn, may result from enhanced leaching from mosses driven by higher precipitation during that year. Similar findings were reported by others in European areas [35,36]. To minimize the impact of meteorological variations on pollutant deposition rates, sampling in future surveys should ideally be conducted under comparable climatic conditions across different years.

### 3.3. Nitrogen—Spatial Patterns in 2022 and Temporal Trends Since 2017

Based on Pitcairn et al. [19] estimation, the nitrogen concentrations in mosses from Yancheng in 2022 indicated that the atmospheric nitrogen deposition was below 20 kg N ha^−1^ yr^−1^. Such quantitative evaluations provide crucial scientific evidence for understanding the environmental impacts of atmospheric nitrogen deposition in Yancheng, particularly in coastal ecosystems like the Yellow Sea Wetlands. The spatial distribution of atmospheric nitrogen deposition is influenced by several factors, including agricultural practices, energy use, and meteorological conditions (e.g., precipitation and wind direction) [37,38]. In 2022, agricultural activities remained the primary source of atmospheric nitrogen deposition in Yancheng (Figure 5, factor 4). Furthermore, there was no significant change in the distribution of agricultural activities compared to 2017. Therefore, it is likely that meteorological conditions contributed to the observed shift in nitrogen deposition distribution. In 2022, Yancheng experienced several extreme weather events, including heavy rainfall, strong winds, convective storms, and typhoons [39]. The convective weather system moving from north to south may have caused nitrogen pollutants to spread from the central region to the south, leading to an increase in nitrogen deposition in these areas. This phenomenon mirrors observations in Europe, where upwind areas redistributed nitrogen from industrial and agricultural hotspots to downwind regions, as documented in airborne nitrogen deposition monitoring campaigns around the Baltic Sea [40]. A study in Switzerland demonstrated a significant correlation between N concentrations in mosses and N loads in precipitation [41]. Nitrogen deposition monitoring in the Gulf of Mexico indicated that 30% of the annual average nitrogen deposition was concentrated in just 9 days of extreme precipitation throughout the year [42]. During the summer of 2022, short-duration, intense rainstorms in the northern and southern parts of the city could have washed nitrogen pollutants from the atmosphere onto the surfaces of moss, significantly increasing the concentration of nitrogen deposition.

### 3.4. Source Appointment of Trace Metals and Nitrogen in 2022

The significant correlations between elements suggested that they likely shared similar sources of contamination [43]. For example, the strong correlation between Al, Fe, and Mn observed in this study aligned with the findings by Xu et al. [44], and it was believed that natural input was a major source for these elements [45]. A deeper PMF model can search the relationships between elements and their pollution sources further. Factors 1, 2, 3, 4, and 5 were predominantly loaded on Al/Fe/Mn, Pb/Zn, Cu/Ni/Cr, Co/Zn/V/N, and Cr/Ni/Co/V, respectively (Figure 5).

Al and Fe are among the most abundant elements in the crust of Earth, and several studies have indicated that wind-blown dust from local soils was a major source of Al, Fe, and Mn in the atmosphere [46,47]. The elemental ratios of Al, Fe, and Mn in the crust are typically stable, with an Al/Fe ratio averaging around 1.46 and a low Mn/Fe ratio, usually about 0.02 [48]. In this study, the calculated average Al/Fe and Mn/Fe ratios were 1.13 and 0.03, respectively, which were in close alignment with the standard values found in the crust. This strongly suggested that these elements predominantly originated from natural sources. As a result, Factor 1 was identified as a natural source.

Vehicle emissions have been identified as one of the main sources of atmospheric Pb [49]. Although leaded gasoline was phased out in China in 2001, residual Pb from its use continues to re-enter the atmosphere through road dust [50]. Moreover, the elevated levels of Pb and Zn could also be linked to tire wear, brake pad abrasion, and additives in lubricating oil [51,52]. Yancheng, a major hub for the automobile industry in China, has a well-developed local automotive market, with residents heavily relying on cars for daily transportation. This dependence on automobiles likely contributed to the atmospheric presence of Pb and Zn. Notably, the spatial distribution of Pb and Zn bioaccumulation in mosses demonstrated high correspondence with major transportation infrastructures, including highways and railway stations (Figure 2h,j,k). Therefore, Factor 2 was associated with traffic emissions.

Cu, Ni, and Cr serve as critical components in stainless steel manufacturing and advanced alloy engineering. High-temperature metallurgical operations, including smelting and casting, release airborne particulates enriched with these metals through vapor condensation and mechanical abrasion [53]. Additionally, industrial activities such as electroplating, metal surface treatment, and thermal spraying contributed to the emissions of these metals [54,55]. High concentrations of Ni, Cr, and Cu were observed in moss samples from the northern region of Yancheng, where the metal smelting and processing industries are particularly prominent. Notably, Xiangshui County, in the northern part of Yancheng, is home to a well-developed stainless steel sector, with a fully integrated industrial chain encompassing nickel–iron smelting, stainless steel production and processing, research and development, workforce training, and marketing. During metal smelting and processing activities, gaseous contaminants, including Ni, Cr, and Cu, were emitted and subsequently accumulated in surrounding mosses through atmospheric deposition. Therefore, Factor 3 was identified as primarily originating from industrial activities.

In China, the elements Co, Zn, V, and N are integral to agricultural production. For instance, Co is commonly used in phosphate and trace element fertilizers, while Zn is an active ingredient in certain bactericides applied to food and cash crops [56,57]. V is incorporated into phosphate fertilizers and certain pesticide formulations. Chronic application of these agrochemicals drives the progressive accumulation of V in agricultural soils, while irrigation practices utilizing V-contaminated wastewater synergistically elevate soil V concentrations [58]. These metals are released into the atmosphere as dust or aerosols during fertilization and production, contributing to atmospheric pollution. Nitrogen, essential for plant growth, plays a vital role in boosting crop yields, and as a result, nitrogen fertilizers are extensively used in agriculture. However, excessive nitrogen fertilization can lead to increased ammonia volatilization, contributing to atmospheric nitrogen pollution [59]. China is the top user of nitrogen fertilizer in the world, contributing to over 22% of its global usage in agricultural production [60]. Additionally, animal manure is considered another significant source of Co, Zn, and N in the atmosphere [61]. Therefore, Factor 4 was identified as an anthropogenic source resulting from agricultural activities.

Previous studies have indicated that human activities, particularly the combustion of fossil fuels such as coal and heavy oil, have significantly increased atmospheric Cr, Ni, Co, and V emissions. In fact, the V emissions from coal combustion in China accounted for 50.55% of the global total, with electric power plants being the largest contributors [62]. The combustion of coal/heavy oil releases Ni and Co, while Cr is enriched in coal fly ash [63]. In this study, the high concentrations of Cr, Ni, Co, and V were found in the northern Yancheng regions known for their concentration of chemical parks and industrial zones. These industries rely on coal-fired or heavy oil-fired boilers and power generation equipment for heating and electricity. Additionally, several small to medium-sized coal-fired power plants still operate in these areas. Consequently, Factor 5 was attributed primarily to fuel combustion from coal and heavy oil.

Overall, fuel combustion (Factor 5) was apportioned as the largest percent contribution (22.71%) for the trace metals and nitrogen in mosses in Yancheng, followed closely by natural sources (Factor 1, 21.4%), agricultural activities (Factor 4, 19.37%), industrial activities (Factor 3, 18.68%), and traffic emissions (Factor 2, 17.84%). We noted that anthropogenic sources were predominant, contributing approximately 78.6% to the metal and nitrogen concentrations in the mosses from Yancheng. Compared to 2017, the sources of atmospheric trace metal and nitrogen contamination in Yancheng did not undergo significant changes by 2022. However, the contribution from agricultural activities significantly increased from 2% to 19.37%. This shift is likely attributed to the inclusion of nitrogen in the source apportionment analysis using the PMF model in 2022. Since atmospheric nitrogen in Yancheng primarily originated from agricultural activities, its inclusion elevated the overall contribution of agriculture to total pollution sources.

## 4. Materials and Methods

### 4.1. Description of the Study Area

Yancheng is a coastal city in eastern China, situated between 32°34′–34°28′ N and 119°27′–120°54′ E (Figure 6a). The entire area features a flat landscape and serves as a transition zone from the north subtropical to the warm temperate climate, with a mean annual temperature of 15.8 °C and an average annual precipitation of 1346 mm. It exhibits a distinct monsoon climate, characterized by prevailing northerly winds with cold conditions in winter and southerly winds with hot weather in summer [64]. Notably, the city is home to the Yellow Sea-Bohai migratory bird habitat, the only natural world heritage site in Jiangsu Province, China. It also boasts excellent transportation infrastructure, including a comprehensive network of highways, railways, air travel, sea routes, and inland shipping. According to reports from the Yancheng Ecology and Environment Bureau, the concentration of PM_2.5_ in the air from 2018 to 2021 was 41, 39, 33, and 27.7 μg/m^3^, respectively, showing a steady decline. However, it still remains significantly higher than the national primary standard of 15 μg/m^3^ [65].

### 4.2. Sampling Procedure

The mat-forming pleurocarpous bryophyte *Haplocladium microphyllum* (Hedw.) was selected for this study based on principal considerations: first, it is ecologically dominant in natural habitats and widely distributed in Yancheng and other regions across China; second, it has been shown to exhibit no significant correlation between trace metal concentrations in its tissues and soil concentrations, with the metal levels in its tissues primarily reflecting atmospheric sources [66]; and third, to ensure methodological consistency and facilitate cross-temporal comparisons, this study utilized the same moss species as the 2017 biomonitoring campaign conducted in Yancheng [17]. Sampling and sample preparation followed the guidelines outlined in the European moss survey [67]. Mosses were collected from 67 evenly distributed sampling sites across Yancheng during July and August 2022 (Figure 6b). The geographic coordinates of these sites were mostly consistent with those from 2017, except for site No. 57, which was not sampled due to road construction, resulting in one fewer site in 2022 (Table S1). Wherever possible, sampling sites were situated a minimum of 300 m away from major roads, highways, villages, and industrial facilities, at least 100 m from secondary roads and buildings, and 3 m beyond the nearest canopy projection. At each site, a composite sample was taken as a representative site sample, consisting of five sub-samples collected within a 50 m × 50 m area. Sub-samples were gathered with a plastic spatula in open areas from brick or rock surfaces and then transferred into tightly closed paper bags for transporting to the laboratory. In the laboratory, coarse contaminants were carefully removed while selecting the green to brown-green part of moss sprouts using plastic tweezers. These segments represent the last three years of moss growth, thereby mitigating the influence of tissue aging on their cation absorption and retention capabilities [67]. The selected green and brown-green segments were dried at 40 °C until reaching a stable mass and then ground into a powder with liquid nitrogen for further analysis.

### 4.3. Elemental Analysis and Quality Assurance

Hotplate digestion was employed to fully digest the moss samples for trace metal analysis, following established protocols from Zhou et al. [17]. About 0.3 g of each moss sample was placed in a 50 mL conical flask and subjected to digestion for 48 h using 10 mL of a mixed acid solution (comprising 2 mL of 70% HClO_4_ and 8 mL of 71% HNO_3_). The resulting mixture was then heated on an electric hot plate at 195–210 °C until it turned clear. After cooling, 2 mL of diluted nitric acid (HNO_3_:H_2_O = 1:1) was added, and the solution was reheated until all white fumes had dissipated. Once cooled again, the volume was adjusted to 25 mL with deionized water to complete the preparation. The levels of Al, Co, Cr, Cu, Fe, Mn, Ni, Pb, Zn, and V were quantified using inductively coupled plasma–atomic emission spectrometry (ICP-AES) (Optima 8300 ICP, Perkin Elmer, Waltham, MA, USA). For each moss sample, triplicate digests were conducted with subsequent triplicate analytical measurements for each digest. Metal contents were expressed in mg kg^−1^ (dry weight basis), while N content was determined using the Kjeldahl method and presented as a percentage of dry weight. 

The analytical quality assurance for ICP-AES and Kjeldahl analysis was implemented through replicated testing of experimental samples alongside parallel measurements of certified moss reference materials (M2 and M3; *Pleurozium schreberi*) supplied by the Natural Resources Institute of Finland. M2 represented moss from polluted areas affected by emissions, while M3 corresponded to samples from background environments [68]. The method detection limits calculated as 3 SD of the procedural blanks were 0.05 mg/kg for Al, 0.001 mg/kg for Co and Mn, 0.002 mg/kg for Cr, Cu, Zn, and V, 0.10 mg/kg for Fe, 0.003 mg/kg for Ni, 0.05 mg/kg for Pb, and 0.02% for N. The recommended and measured values for metal and N concentrations in these reference materials are shown in Table S2. Recovery of the measured concentrations ranged from 88% to 112%, indicating the accuracy of our measurements.

### 4.4. Mapping

Spatial distributions of metal and nitrogen concentrations in mosses were generated using the ordinary kriging interpolation tool in ArcGIS 10.2 (Esri Corporation, Redlands, CA, USA). The kriging interpolation utilized mean moss concentration data from adjacent sampling points combined with the structural features of the variogram to provide optimal linear estimates of regional variables across unsampled areas, establishing spatially continuous surfaces. Prior to interpolation, data exploration was performed to assess whether the raw data followed a normal distribution, identify and remove outliers, and conduct trend analysis. Subsequently, optimal variogram model parameters were calibrated and validated through cross-validation to assess prediction accuracy.

### 4.5. Statistical Analysis

The contamination factor (CF) was used to assess the level of metal contamination. It was calculated by dividing the median concentration of each element (*C_i_*) by its corresponding background concentration (*BC_i_*) [69].

(1) $$CF=C_{i}/{BC}_{i}$$

The background concentration (BC) of an element generally refers to the value of the element in an uncontaminated area within the studied region; however, the delineation of uncontaminated area is difficult due to widespread contamination. Carballeira and Fernandez [70] proposed that the BC of an element should be considered as the concentration of the element in an area of the studied region that, although impacted by anthropogenic activities, remains relatively well-preserved. To assess elemental BCs in terrestrial mosses for this study, a sub-population exhibiting minimal mean concentrations with moderate variability (coefficient of variation, CV < 40%) was designated as representing uncontaminated sites [69,70], and BC was calculated as [mean − 2 STDEV] through iterative removal of maximum values until achieving CV < 40%. Contamination intensity was stratified into six tiers per Shakya et al. [18]: no contamination (CF < 1), suspected contamination (1 ≤ CF ≤ 2), slight contamination (2 < CF ≤ 3.5), moderate contamination (3.5 < CF ≤ 8), severe contamination (8 < CF ≤ 27), and extreme contamination (CF > 27).

Spearman correlation analysis was performed using R (version 4.3.3) to assess inter-element association magnitudes. Statistically significant correlations (*p* < 0.05) indicated potential similar sources among co-associated elements.

The Positive Matrix Factorization (PMF) model was applied as an extension of the Spearman correlation analysis to quantify the relative contributions of metal sources to the moss samples. This model decomposes environmental data matrices into source profiles and contribution matrices [15]. The implementation adhered to EPA PMF 5.0 specifications with parameterization conducted through the PMF 5.0 computational platform:

(2) $$x_{ij}=\sum_{k=1}^{p}g_{ik}f_{kj}+e_{ij}$$

where *x_ij_*, *g_ik_*, and *f_kj_* represent the metal *j* concentration in sample *i*, the relative contribution of source *k* to sample *i*, and the metal *j* content in source *k*, respectively. *e_ij_* denotes the residual value for metal *j* in sample *i*. The objective function *Q* reaches its minimum value through the following calculation:

(3) $$Q=\sum_{i=1}^{n}\sum_{j=1}^{m}{\left(\frac{x_{ij}-\sum_{k=1}^{p}g_{ik}f_{kj}}{u_{ij}}\right)}^{2}$$

where *u_ij_* represents the measurement uncertainty for metal *j* in sample *i*. When metal concentrations fall below the method detection limit (*MDL*), this uncertainty is determined through the following expression:

(4) $$Unc=\frac{5}{6}\times MDL$$

When metal concentrations surpass the *MDL* threshold, the associated uncertainty is calculated using the following:

(5) $$Unc=\sqrt{{\left(U_{rel}\times C\right)}^{2}+(0.5\times {MDL)}^{2}}$$

where *U_rel_* denotes the relative error fraction (assigned 0.1 in this study), and C represents the measured metal concentration.

To evaluate temporal trends in Yancheng between 2017 and 2022, the Mann–Whitney U test was employed to determine statistical significance between comparative datasets. Statistical hypotheses were examined at α = 0.05, with the null hypothesis stating no significant inter-dataset variation.

## 5. Conclusions

This study investigated the concentrations of trace metals (Zn, Cu, Ni, Co, Fe, Mn, V, Al, Pb, Cr) and nitrogen in moss samples collected from Yancheng in 2022. It analyzed their distribution patterns and temporal trends since 2017, as well as explored their potential sources using the PMF model. The results indicated that northern and southwestern Yancheng continued to experience high levels of trace metal pollution, while areas with elevated nitrogen pollution have shifted from central Yancheng to the northern, southwestern, and eastern coastal regions. The concentrations of trace elements (Zn, Cu, Ni, and Cr) and contamination levels of Pb and Cu have risen in the 2022 Yancheng moss survey. Meteorological factors, including precipitation patterns and extreme weather events, may partly contribute to the observed differences between the 2017 and 2022 data. To reduce the impact of meteorological variability on metal and nitrogen deposition rates under wet and dry conditions, sampling should ideally be carried out under consistent climatic conditions each year. The PMF model analysis has proven to be an effective tool for classifying and identifying the potential factors influencing air pollution, and it quantified that anthropogenic sources contributed approximately 78.6% to the metal and nitrogen content in 2022 Yancheng mosses. This study emphasizes the effectiveness of moss biomonitoring in assessing atmospheric deposition and provides valuable insights into the ongoing environmental challenges faced by Yancheng. Continued monitoring and source apportionment analyses are crucial to understanding the dynamics of atmospheric pollution and mitigating its impact on both ecosystems and human health.

## Figures and Tables

**Table 1:** Descriptive statistics of metal concentrations (mg/kg) and nitrogen content (%) in Yancheng moss samples (n = 67) collected in 2022.

Elements	Zn	Cu	Ni	Co	Fe	Mn	Al	Pb	Cr	V	N
Min	102	1.09	3.16	0.33	1338	60.7	1060	2.88	8.73	3.88	0.85
Max	3900	258	70.6	8.28	13,166	421	14,153	216	114	22.6	3.81
Median	835	36.0	14.4	3.85	4263	160	4817	18.1	34.1	8.76	1.61
Mean	995	42.2	17.0	4.01	4517	171	5416	29.3	39.4	9.66	1.67
SD	701	34.1	11.3	1.38	2300	72.5	2642	36.9	23.7	3.74	0.53
coefficients of variation (CVs, %)	70	81	66	34	51	42	49	126	60	39	32

**Table 2:** The comparison of metal median concentrations in 2022 Yancheng mosses with respective those in Mongolia and Norway (mg/kg, DW).

Elements	Yancheng, China (Current Work) n = 67		Mongolia [9] n = 39		Norway [9] n = 228	
	Median	Range	Median	Range	Median	Range
Moss species	*Haplocladium microphyllum*		*Hylocomium splendens* *Pleurozium Schreberi*		*Hylocomium splendens* *Pleurozium Schreberi*	
Zn	835	102–3900	47.1	26.2–69.6	31.1	8.07–409
Cu	36.0	1.09–258	-	-	4.20	1.80–374
Ni	14.4	3.16–70.6	3.88	1.50–11.8	1.09	0.44–547
Co	3.85	0.33–8.28	-	-	-	-
Fe	4263	1338–13,166	4310	1960–11,000	307	78.4–8125
Mn	160	60.7–421	-	-	-	-
Al	4817	1060–14,153	8070	3510–19,300	461	98.4–3048
Pb	18.1	2.88–216	10.0	4.41–23.1	1.58	0.28–22.2
Cr	34.1	8.73–114	8.70	3.90–22.0	0.66	0.19–16.9
V	8.76	3.88–22.6	-	-	1.19	0.31–14.4

**Table 3:** The contamination factors (CFs) and contamination classification.

Elements	Zn	Cu	Ni	V	Co	Fe	Mn	Al	Pb	Cr
CF (2022, n = 67)	7.45 ^2^	6.50 ^2^	6.87 ^2^	4.42 ^2^	3.20 ^1^	5.14 ^2^	4.88 ^2^	5.50 ^2^	11.23 ^3^	6.35 ^2^
CF (2017, n = 68)	4.97 ^2^	3.50 ^1^	5.10 ^2^	4.36 ^2^	—	4.95 ^2^	4.68 ^2^	—	7.98 ^2^	5.68 ^2^

Note: ^1^ Slight contamination; ^2^ moderate contamination; ^3^ severe contamination.

## Data Availability

Data are contained within the article or Supplementary Material.

## Supplementary Materials

The following supporting information can be downloaded at: https://www.mdpi.com/article/10.3390/plants14091315/s1, Table S1 Site-specific geographic coordinates and elemental concentrations in mosses from Yancheng in 2022; Table S2: Recommended and measured values for the concentrations of metals (mg kg^−1^, DW) and nitrogen (%, DW) in moss reference material M2 and M3; Table S3: Total variance of principal component analysis (PCA) for elements in mosses from Yancheng, in 2022; Table S4: Distribution of model scaled residuals for 4 (a) or 5 (b) factors; Table S5: Summary of displacement (DISP) diagnostics by run for Yancheng moss data; Figure S1: Seasonal wind direction frequency patterns in Yancheng, 2022. (Spring: Mar–May|Summer: Jun–Aug|Autumn: Sep–Nov|Winter: Dec–Feb) (Data source: URL https://tianqi.2345.com/); Figure S2: Meteorological trends in Yancheng: Precipitation and temperature differences between 2017 and 2022 (Data source: Jiangsu Provincial Bureau of Statistics, China https://tj.jiangsu.gov.cn/). References [68,71] are cited in the supplementary materials.
