# Supplementary material for: Mosses as Biomonitors of Atmospheric Trace Metal and Nitrogen Deposition: Spatial Distribution and Temporal Trend in Yancheng, China
Source: Plants (Basel). 2025 Apr 26;14(9):1315. doi: 10.3390/plants14091315 (PMC12073996; doi:10.3390/plants14091315)
Supplement: Supplementary file 1 [file plants-14-01315-s001.zip › plants-3497489-supplementary.pdf]

Supplementary materials for

Mosses as biomonitors of atmospheric trace metal and nitrogen deposition: Spatial distribution and temporal trend in Yancheng, China

Xiaoli Zhou [1,2+], Jing Li[1+] , Peng Yan[1], Nana Lu[1], Linyu Lu[1], Qian Ni[1], Junrong Zhang and Yanming Fang [3,*]

[1] School of Marine and Biological Engineering, Yancheng Teachers University, Yancheng, 224002, China; zhouxiaoli0404@163.com; 15251897336@163.com; YanPengsyjsq@163.com; lunana0621@163.com; 15251069766@163.com; 19518893186@163.com; 13270045660@163.com;

[2] Northeast Institute of Geography and Agroecology, Chinese Academy of Sciences, Changchun, 130102, China;

[3] Co-Innovation Center for Sustainable Forestry in Southern China, College of Life Sciences, Nanjing Forestry University, Nanjing 210037, China;

+ These authors contributed equally to this work.

* Correspondence: jwu4@njfu.edu.cn;

Table S1 Site-specific geographic coordinates and elemental concentrations in mosses from Yancheng in 2022

Sampling Site	Latitude N(°)	Longitude E(°)	Metal Concentrations (mg/kg)										Nitrogen Concentration (%)
			Zn	Cu	Ni	Co	Fe	Mn	Al	Pb	Cr	V	
1	32.84	120.32	489	17.2	ND	ND	3161	122	4787	ND	ND	7.09	2.39
2	32.86	120.24	907	31.3	11.6	0.33	5205	156	8776	45.9	34.7	12.37	1.77
3	32.66	120.15	685	114.2	33.8	1.75	3653	160	4365	37.8	82.1	10.27	1.83
4	32.66	120.54	333	9.9	7.6	ND	3693	109	4817	2.9	19.5	5.76	2.24
5	32.64	120.69	826	36.6	3.2	ND	1368	61	1843	46.7	8.7	6.58	1.83
6	32.73	120.64	582	35.3	10.8	ND	3676	159	5290	216.2	31.2	10.39	1.84
7	32.78	120.53	937	52.0	3.6	ND	1585	78	2078	23.2	16.6	6.37	1.34
8	32.89	120.55	303	36.5	16.9	ND	4478	166	4650	10.5	63.0	11.17	1.71
9	32.76	120.68	1382	48.2	21.7	ND	1819	103	2941	ND	13.3	7.37	2.28
10	32.74	120.84	404	29.0	14.0	0.92	7241	285	11562	18.1	32.0	17.64	1.74

11	32.88	120.75	520	10.5	4.9	ND	2006	67	2543	7.1	14.3	7.36	1.36
12	32.93	120.29	1244	40.4	19.0	3.71	4920	213	6583	81.6	48.2	9.16	1.77
13	33.03	120.47	771	29.4	8.8	3.16	2975	103	3547	38.8	17.9	6.21	1.25
14	33.01	120.64	835	25.7	7.5	3.78	5204	141	6222	8.3	24.9	12.05	2.02
15	32.99	120.81	245	12.5	9.2	3.49	3390	116	3327	5.4	34.4	4.76	1.63
16	33.16	120.63	511	12.7	20.2	5.17	1812	95	1982	12.0	57.2	10.17	1.25
17	33.11	120.41	3900	31.6	13.1	3.28	3939	155	3016	19.5	39.8	5.03	1.61
18	33.08	120.32	395	15.8	7.2	2.43	1888	81	1955	9.0	21.4	6.12	1.13
19	33.22	120.36	559	31.1	9.6	2.76	1794	95	2583	9.1	17.6	6.35	1.48
20	33.34	120.39	665	26.3	18.8	3.45	3624	126	4468	9.2	22.6	6.57	1.53
21	33.42	120.51	916	29.9	5.4	3.00	2928	137	3702	30.9	19.8	12.67	1.35
22	33.38	120.42	479	29.1	19.9	4.68	5639	170	7356	8.5	34.0	8.09	2.58
23	33.22	120.64	572	19.5	5.7	2.96	2616	93	2704	8.6	13.0	5.98	1.56
24	33.45	120.08	944	43.5	9.9	2.76	2408	101	2911	12.3	19.0	13.53	1.26
25	33.38	120.2	512	20.8	10.9	3.63	4438	162	5218	5.6	14.0	4.96	1.81
26	33.25	120.2	1627	42.3	14.4	3.07	3683	119	3350	12.5	36.9	9.48	1.14
27	33.18	119.82	1006	50.5	18.1	4.45	5418	242	6703	50.1	36.5	7.87	1.98
28	33.26	120.04	1174	111.1	22.1	3.48	3394	147	3951	188.4	55.3	6.96	1.34
29	33.29	119.86	3481	56.5	17.5	4.46	4638	161	5291	127.3	31.8	8.76	1.73
30	33.46	119.93	1184	56.1	18.1	4.08	4263	165	5155	47.0	37.3	22.62	1.70
31	33.37	119.76	1837	45.2	7.8	2.50	2234	115	2339	19.5	15.9	3.88	1.18
32	33.5	119.75	1092	48.6	6.3	2.95	2361	95	3477	15.6	16.0	10.21	1.29
33	33.56	119.77	882	42.0	10.8	3.53	3857	142	5953	21.0	34.5	16.34	1.92
34	33.55	119.92	791	24.3	23.7	5.31	2366	92	2374	24.7	54.4	10.19	0.92
35	33.55	119.56	1009	54.8	13.3	4.40	3448	186	4546	17.0	50.4	8.35	1.85
36	33.62	119.64	787	37.6	14.4	3.08	2873	130	3544	12.1	27.2	9.69	1.35

37	33.75	119.65	196	31.3	24.9	5.17	8862	227	11704	9.1	44.3	14.25	1.98
38	33.77	119.78	ND	19.7	50.4	7.17	7574	222	9390	17.0	102.0	16.50	2.23
39	33.84	119.61	1537	65.0	17.3	4.65	5547	244	5451	59.9	42.9	7.39	1.62
40	34.08	119.73	634	21.0	8.5	3.14	3779	155	3926	9.7	24.4	8.60	1.50
41	34.14	119.64	656	40.5	34.3	6.07	4406	247	4606	23.9	78.5	13.63	1.76
42	34.19	119.59	414	26.7	40.7	6.95	5976	211	7722	15.4	91.6	14.41	2.07
43	34.3	119.81	1277	258.5	70.6	8.28	13166	359	6754	40.7	113.8	9.44	3.17
44	34.34	119.79	1428	52.9	29.0	3.78	3683	178	3629	21.2	42.0	11.67	1.84
45	34.21	119.92	165	49.2	27.0	6.04	11653	421	9968	20.9	80.0	ND	ND
46	34.29	120.12	1134	52.3	23.1	4.29	6102	280	7366	22.0	49.9	10.05	1.59
47	34.15	120.25	977	25.7	11.8	3.22	3422	141	4523	9.4	17.9	6.05	2.09
48	34.22	120.05	ND	1.1	33.2	4.49	1338	73	1060	12.2	80.2	10.74	1.10
49	34.13	119.89	2843	45.2	21.9	6.27	10463	356	14153	41.6	58.8	10.83	3.07
50	34.1	120.06	842	36.0	8.2	3.43	4774	175	6413	12.6	27.3	9.39	1.71
51	33.99	119.86	1102	29.0	14.5	3.85	5542	151	7476	9.6	25.9	7.86	1.539
52	33.89	119.9	102	19.8	28.7	5.00	2689	114	3301	22.5	75.7	16.50	1.32
53	33.97	120.02	204	12.6	5.5	2.43	2401	112	3358	9.6	16.3	5.13	0.85
54	34.08	120.24	1414	94.0	14.3	4.67	5191	208	6452	49.6	32.8	13.44	1.55
55	33.34	120.24	2502	32.2	18.2	5.05	8361	290	10942	34.0	46.5	11.66	0.89
56	33.99	120.24	1276	60.6	17.4	5.90	7174	293	8482	44.3	34.1	7.65	1.69
57	33.81	120.34	1530	73.2	15.2	4.14	5495	213	6461	39.4	47.8	18.10	1.42
58	33.76	120.22	671	29.8	9.5	3.67	3361	159	4569	18.8	23.3	6.89	0.90
59	33.82	120.03	844	36.3	14.0	4.00	5564	243	6594	17.8	40.8	ND	1.24
60	33.73	120.03	1777	42.1	11.7	4.79	5403	207	7741	9.1	23.0	7.40	1.32
61	33.68	120.13	812	26.3	12.2	4.42	6111	206	8482	6.7	18.9	12.50	1.60
62	33.55	120.12	716	55.2	6.1	3.56	4453	167	5439	26.5	24.2	7.83	1.61

63	33.51	120.25	1264	61.5	12.8	3.50	4787	209	5434	35.7	47.2	ND	1.72
64	33.47	120.43	748	32.9	13.7	4.16	5876	227	7614	13.5	22.9	7.59	3.81
65	33.69	120.29	785	73.5	21.5	3.87	6093	170	7922	13.9	29.1	8.25	1.96
66	33.64	120.39	1450	44.4	24.2	3.87	4719	185	4695	23.7	93.6	6.69	1.42
67	33.73	120.39	600	22.4	24.0	3.92	4694	178	5318	10.5	45.5	ND	0.85

Note: ND indicates “not detected” (concentration < method detection limit, MDL).

Table S2 Recommended and measured values for the concentrations of metals (mg kg^{-1}, DW) and nitrogen (%, DW) in moss reference material M2 and M3 [1,2]

Elements	M2 (n = 9)		M3 (n = 7)	
	Recommended values	Measured values	Recommended values	Measured values
Al	178±15	182±8	169±10	169±8
Co	0.98±0.06	0.96±0.05	0.115±0.006	0.12±0.01
Cr	0.97±0.17	1.09±0.25	0.67±0.19	0.71±0.07
Cu	68.7±2.5	63.8±6.7	3.76±0.23	3.4±1.0
Fe	262±35	250±13	138±12	132±7
Mn	342±17	330±15	535±30	526±13
Ni	16.3±0.9	15.76±0.50	0.95±0.08	0.89±0.08
Pb	6.37±0.43	6.22±0.22	3.33±0.25	2.94±0.45
Zn	36.1±1.2	37.6±4.9	25.4±1.1	23.9±1.7
V	1.43±0.17	1.42±0.07	1.19±0.15	1.22±0.07
Nitrogen	0.826	0.865	0.671	0.705

Note: Values are mean ± one standard deviation; n = number of samples.

Table S3 Total variance of principal component analysis (PCA) for elements in mosses from Yancheng, in 2022

Component	Initial Eigenvalues		
	Total	% of Variance	Cumulative %
1	4.503	40.939	40.939
2	1.416	12.877	53.817
3	1.395	12.680	66.497
4	0.992	9.023	75.519
5	0.855	7.771	83.290
6	0.695	6.317	89.607
7	0.571	5.192	94.799
8	0.342	3.112	97.911
9	0.120	1.088	98.999
10	0.074	0.675	99.674
11	0.036	0.326	100

Table S4 Distribution of model scaled residuals for 4 or 5 factors

4 factors			5 factors		
Monitoring indicators	Moss samples	Residuals	Monitoring indicators	Moss samples	Residuals
Zn	moss17	7.634	Zn	moss2	3.428
Zn	moss26	5.112	Zn	moss5	-3.21
Zn	moss29	4.722	Zn	moss17	8.558
Zn	moss49	5.379	Zn	moss26	5.923
Zn	moss51	4.117	Zn	moss29	3.712
Zn	moss55	8.319	Zn	moss31	5.016
Zn	moss60	3.325	Zn	moss34	3.049
Cu	moss2	-3.14	Zn	moss44	3.028
Cu	moss3	6.052	Zn	moss49	5.898
Cu	moss8	4.978	Zn	moss51	3.77
Cu	moss19	3.001	Ni	moss9	7.621
Cu	moss28	4.944	Ni	moss20	4.388
Cu	moss43	7.392	Ni	moss25	3.368
Cu	moss45	3.321	Ni	moss44	3.438
Cu	moss49	-3.818	Ni	moss47	3.126
Cu	moss52	3.283	Co	moss43	5.876
Cu	moss54	3.281	Co	moss56	3.504
Cu	moss62	3.832	Fe	moss43	3.637
Cu	moss65	4.494	Pb	moss6	9.877
Ni	moss9	7.615	Pb	moss28	5.608
Ni	moss20	4.658	Cr	moss2	3.005
Ni	moss25	3.842	Cr	moss7	3.018
Ni	moss43	3.557	Cr	moss66	3.672
Ni	moss44	3.475	V	moss2	8.371

Ni	moss47	3.241	V	moss3	3.88
Ni	moss65	3.353	V	moss10	7.806
Co	moss55	4.874	V	moss21	4.318
Co	moss56	3.442	V	moss24	5.134
Fe	moss43	4.325	V	moss30	5.761
Pb	moss2	5.899	V	moss32	3.299
Pb	moss5	7.244	V	moss33	4.572
Pb	moss6	9.474	V	moss57	4.853
Pb	moss12	6.996	N	moss1	3.469
Pb	moss13	6.477	N	moss2	4.799
Pb	moss21	5.334	N	moss4	4.275
Pb	moss26	-3.303	N	moss5	4.332
Pb	moss27	5.403	N	moss15	3.296
Pb	moss28	8.329	N	moss45	-3.396
Pb	moss29	7.467	N	moss46	-3.142
Pb	moss30	4.239	N	moss64	4.117
Pb	moss39	4.431			
Pb	moss49	3.662			
Pb	moss53	5.002			
Cr	moss66	3.449			
V	moss2	6.975			
V	moss10	6.81			
V	moss15	-3.089			
V	moss17	-3.16			
V	moss21	5.207			
V	moss24	5.638			
V	moss25	-3.092			
V	moss30	6.012			
V	moss32	4.214			
V	moss33	4.998			
V	moss43	-7.124			
V	moss52	3.282			
V	moss54	3.278			
V	moss57	5.034			
V	moss61	3.015			
V	moss66	-3.276			
N	moss2	7.449			
N	moss3	7.508			
N	moss10	5.489			
N	moss43	3.188			
N	moss55	-3.09			
N	moss64	5.008			
The proportion of samples with -3 < residual < 3: 91%			The proportion of samples with -3 < residual < 3: 95%		

Note: Total samples: 67 moss samples × 11 monitoring indicators/ moss sample = 737

Table S5 Summary of displacement (DISP) diagnostics by run for Yancheng moss data

0		-0.015	
0	0	0	0
0	0	0	0
0	0	0	0
0	0	0	0

Note: In the first line the first value is an error code: 0 means no error; 6 or 9 indicates that the run was aborted. If this first value is non-zero, the DISP analysis results are considered invalid. The second value is the largest observed drop of Q during DISP.

Below the first line is a table (four lines) which contains swap counts for factors (columns) for each dQ_{max} level (rows). The first row is for dQ_{max} = 4, the second row dQ_{max} = 8, the third dQ_{max} = 15 and the fourth dQ_{max} = 25. If any swaps are present for dQ_{max}=4, the solution has a large amount of rotational ambiguity and caution should be used if interpreting the solution.

dQ_{max} (delta Q maximum): maximum allowed change in Q.

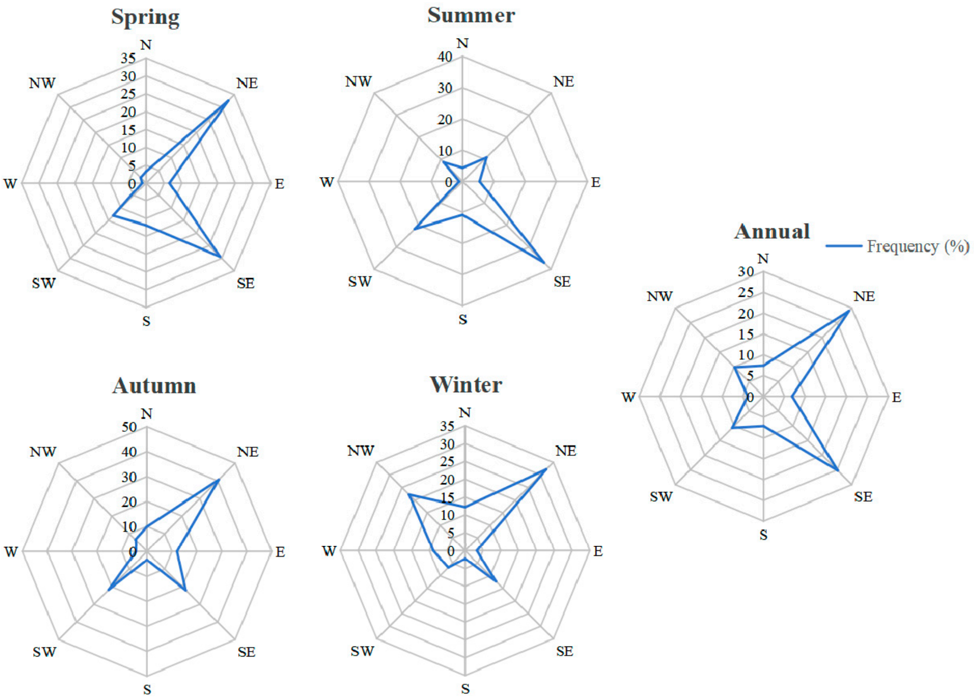

Figure S1 Seasonal wind direction frequency patterns in Yancheng, 2022. (Spring: Mar-May | Summer: Jun-Aug | Autumn: Sep-Nov | Winter: Dec-Feb) (Data source: URL https://tianqi.2345.com/)

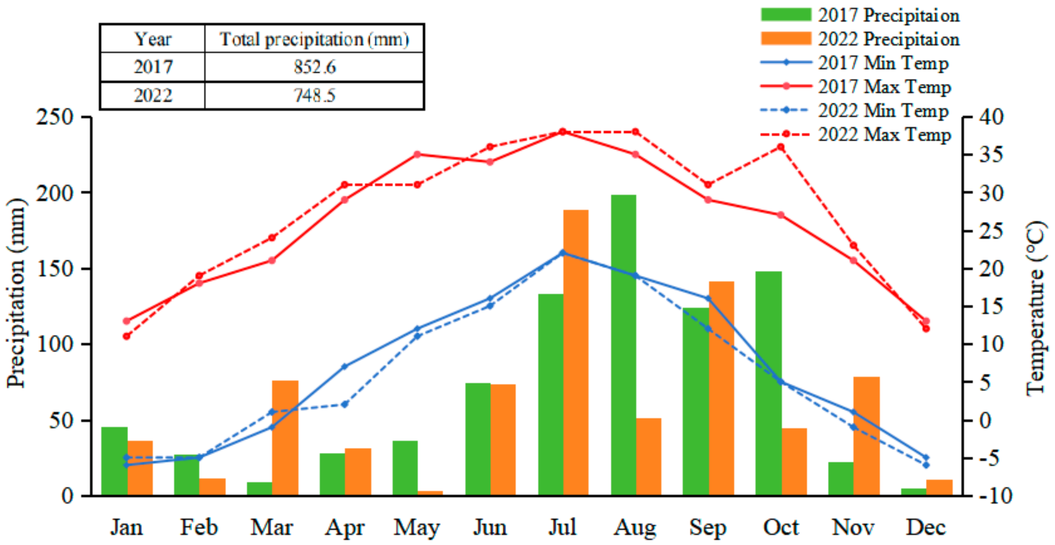

Figure S2 Meteorological trends in Yancheng: Precipitation and temperature differences between 2017 and 2022 (Data source: Jiangsu Provincial Bureau of Statistics, China https://tj.jiangsu.gov.cn/)

References

1. Harmens, H.; Norris, D.A.; Steinnes, E.; Kubin, E.; Piispanen, J.; Alber, R.; Aleksiayenak, Y.; Blum, O.; Coskun, M.; Dam, M.; De Temmerman, L.; Fernández, J.A.; Frolova, M.; Frontasyeva, M.; González-Miqueo, L.; Grodzińska, K.; Jeran, Z.; Korzekwa, S.; Krmar, M.; Kvietkus, K.; Leblond, S.; Liiv, S.; Magnússon, S.H.; Maňkovská, B.; Pesch, R.; Rühling, Å.; Santamaria, J.M.; Schröder, W.; Spiric, Z., Suchara, I.; Thöni, L.; Urumov, V.; Yurukova, L.; Zechmeister, H.G. Mosses as biomonitors of atmospheric heavy metal deposition: spatial patterns and temporal trends in Europe. *Environ. Pollut.* 2010, 158, 3144–3156.
2. Steinnes, E.; Rühling, Å.; Lippo, H.; Mäkinen, A. Reference material for large scale metal deposition surveys. Accreditation and Quality Assurance, 1997, 2: 243–249.
